# The intersection between circadian and heat-responsive regulatory networks controls plant responses to increasing temperatures

**DOI:** 10.1042/BST20190572

**Published:** 2022-06-27

**Authors:** Kanjana Laosuntisuk, Colleen J. Doherty

**Affiliations:** Department of Molecular and Structural Biochemistry, North Carolina State University, Raleigh, U.S.A.

**Keywords:** circadian clock, eukaryotic gene expression, gene regulatory networks, plant signal transduction, temperature sensing, temperature stress

## Abstract

Increasing temperatures impact plant biochemistry, but the effects can be highly variable. Both external and internal factors modulate how plants respond to rising temperatures. One such factor is the time of day or season the temperature increase occurs. This timing significantly affects plant responses to higher temperatures altering the signaling networks and affecting tolerance levels. Increasing overlaps between circadian signaling and high temperature responses have been identified that could explain this sensitivity to the timing of heat stress. ELF3, a circadian clock component, functions as a thermosensor. ELF3 regulates thermoresponsive hypocotyl elongation in part through its cellular localization. The temperature sensitivity of ELF3 depends on the length of a polyglutamine region, explaining how plant temperature responses vary between species. However, the intersection between the circadian system and increased temperature stress responses is pervasive and extends beyond this overlap in thermosensing. Here, we review the network responses to increased temperatures, heat stress, and the impacts on the mechanisms of gene expression from transcription to translation, highlighting the intersections between the elevated temperature and heat stress response pathways and circadian signaling, focusing on the role of ELF3 as a thermosensor.

## Introduction

Extreme temperatures are a consistent challenge for crop growth. As global temperatures increase, research studying how plants adapt to these new climates has likewise grown with the hope of developing crop varieties that can withstand higher temperatures and deliver food security in the face of changing climates.

Exposure to elevated temperatures and heat stress are not synonymous. Heat stress occurs when the temperature reaches a point that damages the cellular components affecting functions such as metabolism, signaling, structure, and transport [1–4]. Molecular changes occur before plants reach this state of heat stress as the plant adapts to the higher temperatures. It would benefit this research area if a distinction was made between plant responses to increased temperature and heat stress. However, this is not easy, no constant threshold temperature exists, and exceeding the optimal growing range does not always invoke cellular damage or death.

**Figure 1.**
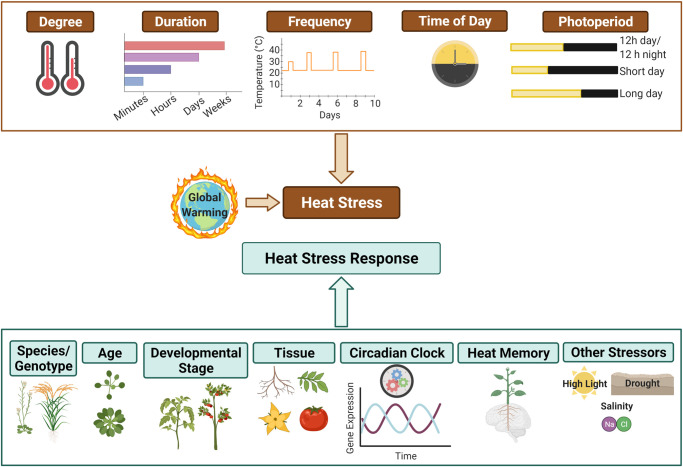
Plant heat stress response is a complex interaction between features of heat stress and internal factors in plants. Heat stress is a combination of heat intensity, duration, and frequency. The time of day and photoperiod when the increased temperature occurs are important factors that impact the severity of heat stress. Internal factors also influence how plants respond to elevated temperatures. These include the genotype, age, developmental stage, tissue, stage of the internal clock, and prior exposure to heat or other stressors. The figure was created with BioRender.com.

Both extrinsic factors related to the temperature event and intrinsic factors reflecting the state of the plant together determine if a plant can withstand a given temperature event (Figure 1). The extrinsic parameters depend on the heat intensity, frequency of heat events, duration, time of day, and time of year [5–12]. Most information on heat stress responses is based on experiments using transient, high temperatures, but field-grown plants tend to experience long-term, moderate heat stress. Prolonged heat stress and heat shock result in different physiological, metabolic, and transcriptomic responses [13]. Even mild heat stress can be damaging over a long period [14]. Moreover, because temperature and air moisture content are inherently linked, the combined effects of elevated temperatures and changes in vapor pressure are critical to identifying how elevated temperatures and heat stress affect actual yields in field conditions [15,16].

Intrinsic, plant-centric parameters that affect heat responses include the plant genotype [17], developmental stage [18–22], prior exposure to heat stress [23–28], the time relative to the plants’ internal circadian clock [5,6,8], and the presence of other stresses (Figure 1) [29–33]. Even the same genotype can have significantly varied tolerance levels and responses to the same temperature increase depending on when the stress occurs [5–7,9,34,35]. The combinations of these extrinsic and intrinsic parameters result in a wide gradient of plant responses. Approaches that incorporate these variables have enabled new insights into heat stress tolerance. We review how time of day and circadian networks overlap with elevated temperature and heat stress responses through multiple levels of gene expression and the role of ELF3, a circadian clock component, in temperature sensing.

## Interactions between the circadian clock and elevated temperatures

**Figure 2.**
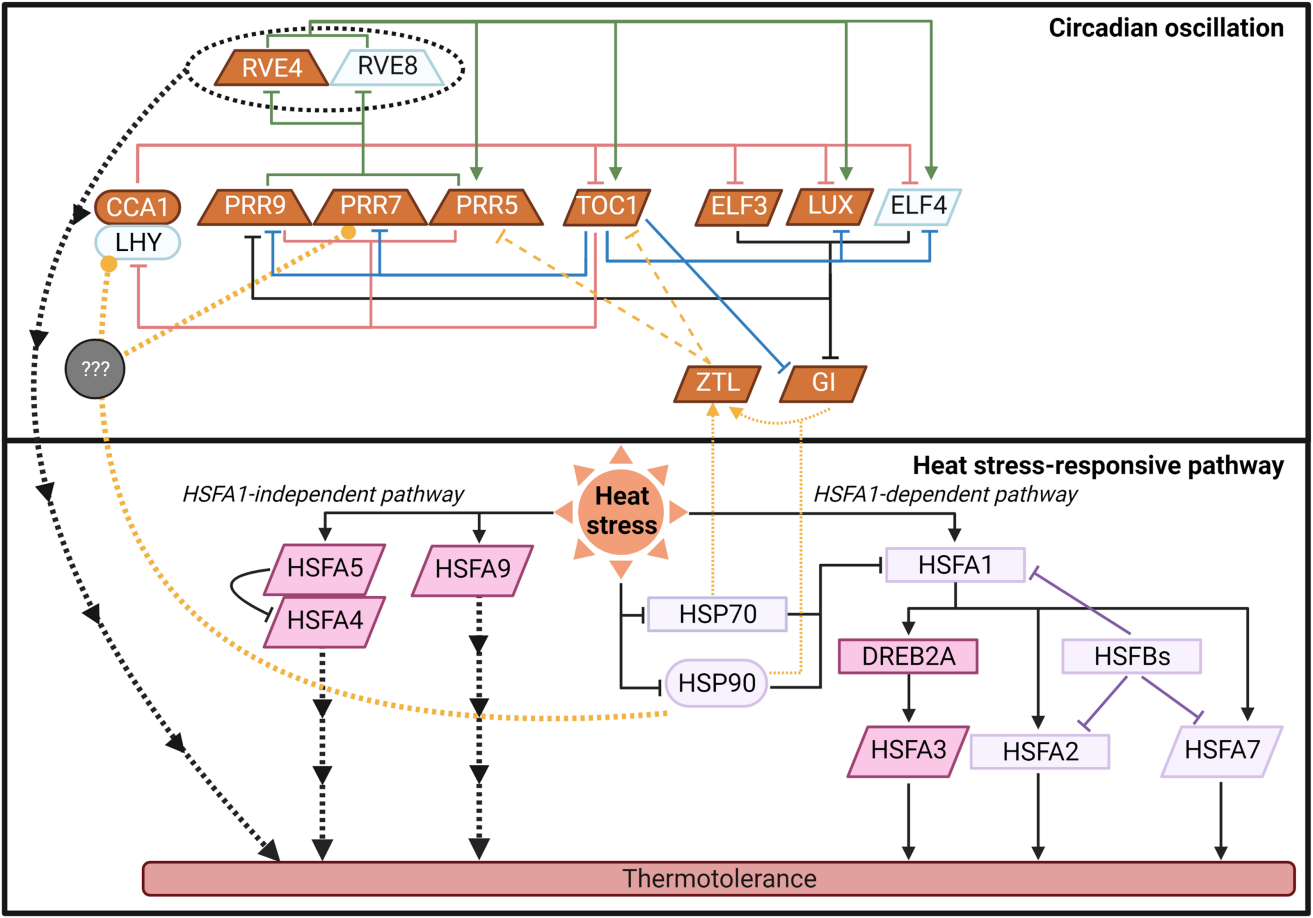
Crosstalk between the circadian clock and heat stress-responsive pathways. The diagram of circadian oscillation (top) was adapted from [147]. The colors of the clock components represent their expression level in response to elevated temperatures from Grinevich et al. [5], and Blair et al. [8]. Increased expression is shown in orange and decreased expression in light blue. The heat stress-responsive pathway (bottom) was adapted from [2]. The diagram includes only HSFs with known variation in basal expression between times of day in Grinevich et al. [5]. Colors indicate whether HSFs are targeted by the clock proteins based on the ChIP-Seq data [70–76]. Direct targets of circadian components are shown in dark pink, and light purple means they are not the direct targets. For both sections, the shapes of the text box represent their expression peaks under normal temperature. Ovals, trapezoids, and parallelogram mean high expression in the morning, afternoon, and evening, respectively. Rectangles mean no difference in expression between dawn and dusk. Solid lines indicate that the regulation occurs via transcriptional activation or repression. Dashed lines indicate regulation via post-translational modification. Thin dotted lines represent the regulation through a chaperone function. Thick dotted lines indicate that the regulatory mechanisms are unknown. The figure was created with BioRender.com.

Increased temperatures affect most aspects of plant biochemistry including mRNA stability [36,37], splicing [38–42], translation [43–45], post-translational modifications [46–51], and protein degradation [52–56]. These pathways are themselves circadian-regulated (Figure 2) [52,53,56–63]. Thus, there is significant cross-regulation between the response to elevated temperatures, heat stress and circadian clock networks and their downstream targets. Such interactions between heat stress response and circadian-regulatory networks are supported by the fact that thermotolerance is gated by the time of the day when the elevated temperature occurs. In controlled environments, plants are more sensitive to elevated temperatures at dawn than at dusk [5,9,64]. About 68% of heat-responsive genes are gated by the time of day [5]. This large overlap suggests that, in part, temporal sensitivity of thermotolerance is due to interactions between the circadian-regulatory and heat stress response networks.

Conversely, elevated temperatures also affect the expression of circadian clock components (Figure 2) [8,65]. This complex coordination between the plant circadian clock and elevated temperature responses indicates that considering the effects of both factors is essential to acquiring a predictive understanding of plant responses to increased temperatures.

### Time of day and circadian regulation of heat stress response networks

#### Daily expression of HSFs

The transcriptional response to elevated temperatures centers around the roles and regulation of Heat Shock Factors (HSFs) and Heat Shock Proteins (HSPs). In response to heat stress, type-A HSFs (HSFA1s) trimerize and bind Heat Shock Elements (HSEs) in the promoters of their target genes. This results in the induction of their targets, such as the transcription factor DEHYDRATION-RESPONSIVE ELEMENT BINDING PROTEIN 2A (DREB2A) and co-activator MULTIPROTEIN BRIDGING FACTOR 1C (MBF1C) [2,66,67].

Upon activation, HSFA1s trigger a gene expression cascade that results in new proteins that change the cellular composition improving tolerance. Critically, the network the response to elevated temperature traverses depends entirely on the molecules available at the time of the stress. This molecular landscape of the specific gene expression activity, chromatin state, mRNA abundance, protein composition, and metabolite levels varies significantly depending on the time of the increased temperature event (Figure 1). For example, in Arabidopsis and rice (*Oryza sativa* L.), the mRNA levels of *HSFs*, *HSPs*, and other regulators of heat stress vary significantly depending on the time of day [5,68] (Figure 2). In non-heat stress conditions in Arabidopsis, 17 out of 21 *HSF* genes show rhythmic expressions under diel conditions [5,69]. But the patterns of expression vary. For example, *HSFA1a* is highly expressed in the morning while *HSFA1b*, *1d*, and *1e* have a peak expression in the evening. A few *HSF* genes and some of the *HSF* targets, such as *DREB2A*, are the direct targets of the circadian clock components proteins (Figure 2) [70–76]. For example, *HSFA3* is a CCA1 target [5,8], and its expression is altered in cca1/lhy double mutant under elevated temperature [8]. The *HSFs* and other heat-response regulators that show robust rhythmic expression could be responding to diel changes in light and temperature or indirectly regulated by the clock components via unknown mechanisms.

#### Time of day and circadian regulation of heat shock responses

The circadian clock plays a significant role in heat-responsive gene expression. In response to heat shock, c*ca1/lhy* double mutants and *prr7/9* double mutants have fewer differentially expressed genes and decreased expression-change magnitude. The reduced transcriptional response in these circadian mutants highlights the importance of clock components in the transcriptional response under elevated temperatures [8].

HSFA1s are primary regulators of heat shock responses. Most HSFA1 target genes are up-regulated in response to heat shock at dawn [5]. *HSFA1b* is induced by increased temperatures at dusk but not at dawn, suggesting that *HSFA1b* induction alone is insufficient to regulate most HSFA1 targets [5]. This disconnect between the expression of these regulators and their targets indicates that multiple network strategies for heat stress responses exist and vary depending on the time of day. Critically, these variations in responses can be used to understand how connections between regulators and targets are wired and may explain the specific roles of genes otherwise thought to be redundant. For example, HSFA2 is a target of HSFA1s, is a key regulator of heat acclimation and acquired thermotolerance [26,77], and is up-regulated under elevated temperatures at both dawn and dusk (Figure 2). Although HSFA2 responds to increased temperatures at both times of day, its targets *HSP21*, *HSP22*, *HSP18.2*, and *ASCORBATE PEROXIDASE2 (APX2*) are significantly induced under increased temperatures near dawn yet show substantially lower expression levels when plants are exposed to increased temperatures in the evening [5]. This may indicate required interactions between HSFA2 and other morning responsive HSFA1 targets in the induction of these genes. Another memory protein, HSFA32 [28], also accumulates to higher levels in response to elevated temperatures in the morning than in the evening [5]. The effects of these temporal differences in response on heat stress memory have not been evaluated.

Even though HSFA1 is a master regulator of the heat shock response, ∼40% of heat-responsive genes are HSFA1 independent [9]. The circadian clock proteins RVE4 and RVE8 control the expression of heat-responsive genes in an HSF-independent manner (Figure 2) [9]. As *RVE4* and *RVE8* are highly expressed in the afternoon, this pathway is possibly responsible for regulating thermotolerance during the daytime.

HSFA4, HSFA5, and HSFA9 proteins also function independently of HSFA1, but the downstream targets that confer thermotolerance are less understood (Figure 2) [2]. In Lily (*Lilium Longiflorum*), HSFA4 regulates ROS metabolism to enhance basal thermotolerance [78]. ROS metabolism is also a primary target of the circadian clock [79–81]. Exploring how time alters these transcriptional responses can provide new insights into how the heat stress response networks are wired.

The observed interaction between the circadian clock and heat shock networks persists downstream of transcription. At the global translational level, the time of day gates about one-third of circadian-regulated heat-responsive translatome [65].

### Intersection between heat stress response and circadian networks in gene expression

#### Transcriptional activation

Both A and B classes of HSFs have an affinity for components of the basal transcription machinery [82]. This association with the core transcriptional components provides a mechanism for how increased HSF occupancy at promoters increases RNA Polymerase II (RNAP II) binding and induces transcription of their target genes.

**Figure 3.**
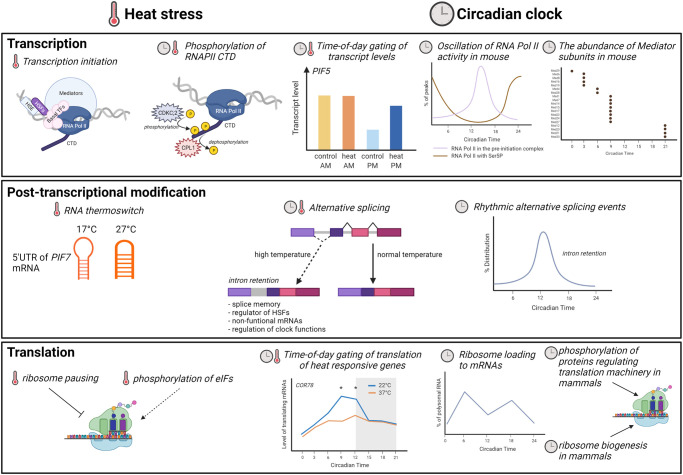
Overlap between heat stress response and circadian-regulatory pathways in gene expression. Elevated temperature responses, heat stress and circadian-regulatory pathways affect multiple regulatory mechanisms controlling gene expression. The expression of PIF5 under heat shock at dawn vs dusk is from Grinevich et al. [5]. The oscillation of RNA Pol II activity and protein abundance of Mediator subunits in mouse are adapted from Koike et al., and Wang et al., respectively [97,98]. The rhythmic alternative splicing events are based on Yang et al. [109]. Ribosome loading to mRNAs and the gating of COLD-REGULATED 78 (COR78) translating mRNA are from Missra et al. and Bonnot and Nagel, respectively [65,148]. The figure was created with BioRender.com.

RNAP II activity is regulated by post-translational modifications of its largest subunit's carboxy-terminal domain (CTD) [83]. Phosphorylation of RNAPII CTD by CYCLIN DEPENDENT KINASE C;2 (CDKC;2) is required for a proper circadian period in Arabidopsis (Figure 3) [84]. Increased temperatures change these RNAP II CTD modifications to alter transcription. In barley anthers, high temperatures result in hyperphosphorylation of the CTD's Serine 5 (Ser5) residue, which alters anther-specific gene expression, leading to an arrest of anther development [85]. In Arabidopsis, RNAP II CTD Ser2 and Ser5 phosphorylation increase during heat shock recovery, coinciding with increased thermotolerant-related gene expression. Mutation of CTD phosphatase-like 1 (CPL1, also known as RCF2/FIERY2) which dephosphorylates the CTD Ser5, reduces the expression of most *HSFs,* and decreases thermotolerance in Arabidopsis [86–88]. This suggests that proper regulation of RNAPII CTD phosphorylation is critical for heat-responsive gene expression. However, CPL1 also dephosphorylates the transcription factor NAC109, which induces the expression of several *HSFs* [89]. CPL1 and its close homolog CPL2 are also part of the plant growth, cell morphogenesis, and other stress response networks, including biotic stress response [86,87,90]. *CPL2* is circadian-regulated and is induced in response to elevated temperatures in the morning but not in the evening [5,91]. In mammalian systems, the core transcriptional machinery, including phosphorylation of Ser5, is circadian-regulated. However, our understanding of the circadian regulation of core transcriptional machinery remains limited in plants. The intersection between the circadian and heat-responsive regulations of the RNAP II activity may explain how some variation in elevated temperature responses is clock-dependent (Figure 3) [92].

HSFA1s’ specificity for target genes may also occur through the recruitment of co-activators. HSFA1s recruit the transcriptional co-activator complex, Mediator, to the promoters of the heat-inducible genes [93]. However, not all HSFA1 target genes were equally affected in plants with disrupted Mediator complex. For example, loss of the structural hub subunits of Mediator, *med14,* or *med17* resulted in significantly reduced induction of HSFA1 transcription factor targets, such as *DREB2A* and other *HSF*s, but had little effect on the heat-induced expression of *HSP*s [93]. This suggests that Mediator can fine-tune the network responses, even for the targets of the same master regulator, perhaps explaining some of the time of day variation in the induction of HSFA1 targets. The specific downstream network of HSFA1 could be tuned to provide different network responses based on the particular HSFA1 activation mechanism [93] or tailored based on tissue-specific [94] or environmentally induced [95,96] changes in Mediator subunit composition. Loss of specific mediator subunits reveals distinct roles in regulating which genes are expressed under heat stress in Arabidopsis, supporting the idea that different mediator compositions could modulate unique targets [96]. In particular, loss of MED16 resulted in misregulation of many heat-responsive genes, consistent with the role of this subunit in heat responses in *S. cerevisiae* [96]. In plants, most experimental evidence of potential mediator subunit composition differences is based on changes in mRNA levels. However, in mammalian systems, variation in the Mediator subunit composition has been shown at the protein level, including variation based on the time of the day [97]. In addition, the recruitment of basal transcription factors and activation of RNAP II vary across the time of day in mammals (Figure 3) [98]. Yet the complete transcriptional landscape over the time of the day in plants has not been examined. As the magnitude of transcript levels of heat-responsive genes is influenced by the time plants are exposed to elevated temperatures (Figure 3) [5,8], studying the fluctuation of transcription machinery during the day would provide a better insight into the time-of-day gating of basal thermotolerance in plants.

#### Post-transcriptional modification

The secondary structure of single-stranded mRNA is important for polyadenylation, splicing, translation, and turnover [99]. Bacteria have RNA thermometers, RNAs with temperature-sensing sequences, to control translation efficiency in response to temperature changes [100]. Unfolded RNA thermometers increase translation efficiency under heat stress [100]. In Arabidopsis, the expression of *PHYTOCHROME INTERACTING FACTOR7 (PIF7*) and *HSFA2* are proposed to be regulated via an RNA thermoswitch [101]. PIF7 is a bHLH transcription factor regulating thermomorphogenesis [102], suggesting that RNA structure could be another mechanism to temporally regulate thermoresponsive growth. In rice, heat shock unfolds mRNA, promoting mRNA degradation [36]. Some evidence for circadian regulation of RNA stability exists in Arabidopsis [60]. For example, *CCA1* is a target for m6A RNA methylation in response to blue light, which accelerates its degradation [103]. However, most plant studies that examine either elevated temperature responses, circadian regulation, or both measure only steady-state RNA levels without distinguishing between transcriptional activation and mRNA degradation. Therefore, this area needs further investigation to determine if it contributes to the temporal variation in increased temperature responses.

Alternative splicing (AS) is a way to generate transcript variants from a single gene. These variants can affect mRNA stability or result in different protein isoforms. Heat stress induces AS in Arabidopsis, wheat [38], tomato pollen [39], grape [40], and moss [41]. One hypothesis for this increase in AS is that heat stress increases splicing errors [104]. Heat acclimation, priming plants with a non-lethal temperature that enhances tolerance to severe heat stress [25], also affects AS [105]. For example, in Arabidopsis, primed plants have fewer intron retention (IR) events after severe heat stress than non-primed plants [105]. This result indicates that heat acclimation creates splicing memory, maintaining correct splicing after severe heat stress [105].

AS also regulates the expression of *HSFs* and *HSPs* [42]. Several *HSFs* and *HSPs* undergo AS under heat stress [39,41,106]. In Arabidopsis, heat stress induces AS on *HSFA2,* generating a splice variant *HSFA2-III* which self-regulates *HSFA2* transcription [42]. In lily (*Lilium* spp.), the protein encoded from the heat-inducible splice variant LlHSFA3B-III interacts with the HSFA3 ortholog, LlHSFA3A-I, reducing the accumulation of LlHSFA3A-I, resulting in an attenuation of the heat stress responses [107]. AS is also regulated by the time of day and the circadian clock [108–110]. The core circadian clock genes themselves have temperature-dependent AS, suggesting one mechanism by which the clock and heat stress responses can interact [111–114]. However, variation in splicing after elevated temperatures has not been examined at different times of the day.

#### Translation

Under heat stress, the time of day gates about one-third of the circadian-regulated heat-responsive translatome in Arabidopsis (Figure 3) [65], but the molecular mechanisms underlying this phenomenon are still elusive. In *Neurospora crassa*, circadian control of Eukaryotic Elongation Factor2 (eEF2) [115] and the phosphorylation of Eukaryotic Initiation Factor 2a (eIF2ɑ) [116] result in circadian-regulated translation. In wheat, the phosphorylation state of eIF4A and eIF4B is altered under heat shock while other translation factors, eIF4F, eIFiso4F, eIF2α, and eIF2β remain the same [117]. However, the variation in this phosphorylation in response to elevated temperatures has not been examined at different times of the day. Mammalian studies also show that the circadian clock regulates the translation machinery via ribosome biogenesis and phosphorylation of proteins in initiation and elongation steps (Figure 3, review in [118]). Although translation in plants is circadian regulated [119,120], the specific mechanisms driving clock regulation of translation in plants are unknown [121].

### Regulation of the circadian clock under heat stress

As these multiple levels of interaction between the circadian and elevated temperature response networks affect heat stress responses, they also affect circadian regulation. Elevated temperatures alters the expression of circadian genes (Figure 2) [8,65]. *CCA1*, *LHY*, *PRR7*, and *PRR9* mRNA levels change between 22°C and 37°C. HSPs cooperate with circadian components and impact both heat stress responses and circadian rhythms. HSP90 interacts with ZTL in protein degradation under heat stress [122]. HSP90 and HSP70 work together with GI in ZTL protein maturation [123]. Moreover, HSP90 plays a role in circadian oscillation, interacting with the morning loop [124]. The *hsp90.2.3* mutant has a longer period in temperature cycles (22/16°C) and a phase advance in the late morning. CCA1, LHY, and PRR7, which are part of the morning loop, are required for the period lengthening by HSP90. However, how HSP90 mediates the expression of *CCA1*, *LHY*, and *PRR7* is still unclear [124]. HSFB2b binds the *PRR7* promoter and represses *PRR7* expression in the morning, and temperature compensation, a fundamental characteristic of circadian rhythms, requires HSFB2b [69].

Although the levels of several core clock components are altered between 22°C and 37°C, the daily expression pattern remains unchanged [8,65], suggesting that the circadian clock is functional at 37°C in Arabidopsis. The ability to retain the same period despite the influences of increased temperature is a feature of the circadian clock known as temperature compensation (reviewed in [125]). However, temperature compensation has limits, and outside of these ranges, the disrupted clock could compound the impacts of elevated temperatures and heat stress. Many questions remain about temperature compensation limits: Do limits vary across developmental stages or between genotypes? Does acclimation increase the limits within which the plant can be temperature compensated? What intrinsic and extrinsic factors affect the temperature compensation range? These questions about temperature compensation limits are beginning to be examined in Arabidopsis, and much less is known about these limits in other plant species or in natural conditions.

## The role of the circadian component, ELF3, in temperature sensing

Before activating cellular responses to combat the effects of heat stress, plants must first sense a temperature increase. Multiple temperature sensing mechanisms have been described in plants. These include messenger RNA (mRNA) stability, protein degradation, and histone modifications and have been reviewed recently [126–128]. New research indicates connections between the circadian clock and thermosensing in Arabidopsis through the roles of ELF3 [129–131].

ELF3 is part of the evening complex (EC) with LUX and ELF4. The EC is so named because these components are highly expressed in the evening. As a member of the EC, ELF3 regulates hypocotyl elongation through transcriptional and post-transcriptional control of the basic helix–loop–helix transcription factors, PIF4 and PIF5 [132]. PIF4 and PIF5 positively contribute to light- and temperature-dependent growth, indicating that they integrate light and temperature cues into cellular pathways [133]. In Arabidopsis, increased temperature induces hypocotyl elongation. This thermoresponsive hypocotyl elongation is lost in *elf3-1* mutant plants and can be restored by complementing with exogenous ELF3. Thus, ELF3 is critical for connecting increasing temperature to the physiological response of hypocotyl elongation [129]. ELF3's role in sensing ambient temperature changes extends beyond hypocotyl elongation. ELF3 also functions as a thermosensor in the temperature entertainment of the circadian clock [134].

### ELF3 is a central component of temperature-responsive hypocotyl elongation

ELF3 regulates thermoresponsive hypocotyl elongation transcriptionally and posttranscriptionally. The EC restricts hypocotyl elongation through transcriptional control by repressing the expression of *PIF4* and *PIF5* [132]. Elevated temperatures reduce ELF3 occupancy at the *PIF4* promoter, resulting in PIF4-mediated thermomorphogenesis [135]. In fact, the entire EC complex appears to dissociate from the DNA at higher temperatures [76,136]. However, high ELF4 levels can stabilize EC-DNA binding and overcome this temperature-induced dissociation of the EC from the target DNA [136].

In addition to the transcriptional control of *PIF4* expression through the EC, ELF3 also regulates PIF4 activity [135,137,138]. ELF3 binds the bHLH domain of PIF4 directly, preventing PIF4 from activating its transcriptional targets [139]. Thus, ELF3 regulates thermoresponsive hypocotyl elongation through control of PIF4 at multiple levels.

### ELF3 is a temperature sensor

**Figure 4.**
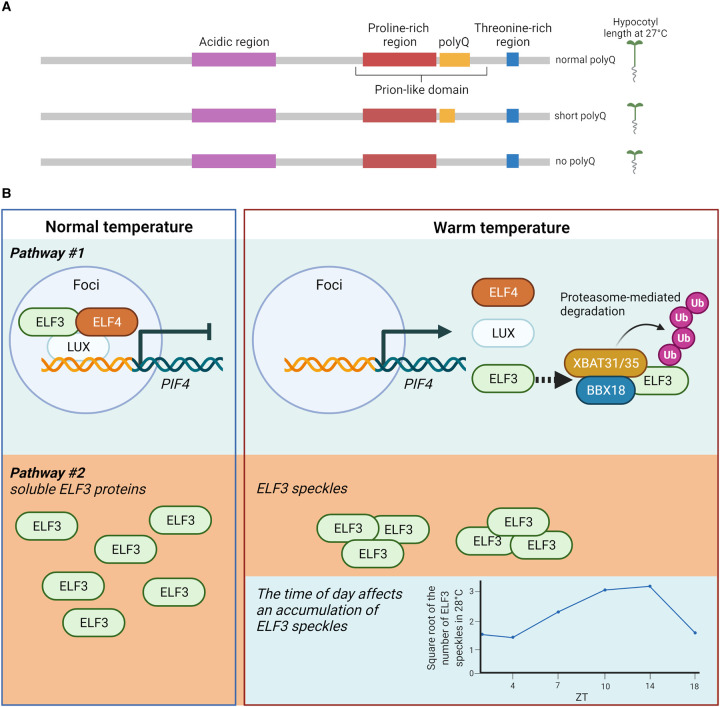
ELF3 functions as a thermosensor in plants. (**A**) ELF3 protein contains 695 amino acids. It can be divided into four regions based on the composition of amino acids: an acidic region (206–320), a proline-rich region (440–540), a threonine-rich region (636–652), and glutamine repeats or polyQ (544–585) [149]. The prion-like domain (PrD) has recently been identified as a thermosensor, and it covers amino acids 430–609 [129]. The length of polyQ inside the PrD determines the degree of thermoresponsiveness. (**B**) The current model of ELF3 in response to warm temperatures in Arabidopsis is based on [129–131,143,144]. Under normal temperature, ELF3 interacts with ELF4 and LUX to form an evening complex in sub-nuclear areas called foci. The EC in the foci then binds to target genes such as *PIF4* to repress their expression. Under warm temperatures, the EC disperses and leaves the foci [131]. The E3 ubiquitin ligase XBAT31/35 binds ELF3 with help from BBX18, targeting ELF3 for proteasome degradation [143,144]. Another model proposes a mechanism to reduce active ELF3 under warm temperatures via the PrD [129]. ELF3 proteins are in a soluble form under normal temperature. As temperatures increase, ELF3 proteins aggregate into speckles. The formation of ELF3 speckles varies throughout the day, as the speckles are more detectable in the afternoon [130]. The figure was created with BioRender.com.

Using thermoresponsive growth to test ELF3's temperature-sensing role, Jung et al. showed that the prion-like domain (PrD) provides part of this temperature-sensing function (Figure 4A) [129]. The PrD varies between species and contains a polyglutamine (PolyQ) tract. Increasing the length of the PolyQ tract increases the thermoresponsiveness in Arabidopsis. Chimeric Arabidopsis ELF3 versions with PrDs from plants from warmer climates reduce thermoresponsiveness [129]. Nevertheless, plants overexpressing ELF3 without a PrD are still thermally responsive, unlike the *elf3-1* mutant, indicating that other features of ELF3 also function in temperature sensing [129].

Jung et al. observed that the PrD affects ELF3 localization in cells [129]. At lower temperatures, ELF3 is soluble and diffused in the cells. However, at higher temperatures, the ELF3 proteins form speckles in root cells and heterologous yeast systems [129]. The formation of these concentrated regions of ELF3 is reversible. When returned to low temperatures, the protein diffuses again. Replacing the Arabidopsis PrD with the equivalent region from *Brachypodium distachyon*, a warm temperature grass that lacks a detectable PrD reduces speckle formation at higher temperatures. This speckle formation is suggested to facilitate remembering warm daytime temperatures during nighttime hypocotyl growth [130]. The nighttime hypocotyl expansion depends on the nighttime temperature *and* the prior daytime temperature [130]. Warm daytime temperatures increase the nuclear level of PIF4 during the day, and the active PIF4 from daytime temperature affects hypocotyl growth at night (Figure 4B) [130,140]. This hysteretic PIF4 pattern requires ELF3 [130]. ELF3 speckle formation correlates with *PIF4* promoter activity; increased ELF3 localization into speckles increases *PIF4* transcription [130]. Warm daytime temperatures reduce speckle formation in hypocotyl cells during the morning and increase speckle formation during the afternoon suggesting connections between the circadian regulation of these components and their temperature sensing and memory functions [130].

Another study observed a different ELF3 localization response under warm temperatures and proposed an alternative mechanism connecting the temperature-sensing ELF3 and downstream physiological changes [131]. In the nucleus, ELF3 and other EC components localize in foci. This localization is important for suppressing EC-target gene expression [141,142]. Ronald et al. observed that warm temperatures disrupt the localization of ELF3 to these nuclear foci in hypocotyl and root cells, and ELF4 is not required for this process (Figure 4B) [131]. At warm temperatures (29°C), ELF3 is no longer localized to the foci and is targeted by E3 ubiquitin ligases, XBAT31 and XBAT35 (Figure 4B). Ubiquitinated ELF3 is degraded by the 26S proteasome removing the repressive effects of ELF3 on PIF4 and allowing hypocotyl elongation [143,144]. The interaction between XBAT31/35 requires B-box domain protein BBX18, which possibly acts as a scaffold protein enhancing the XBAT31/35-ELF3 interaction [143,144]. In this model, heat stress releases ELF3 from the nuclear foci, increasing PIF4 promoter activity. Ronald et al. describe potential experimental differences that could account for the apparent opposite effect of warm temperatures on ELF3 accumulation or diffusion, requiring future studies to distinguish between these mechanisms [129,131].

Intriguingly, *XBAT31/35* are direct CCA1 targets with peak expression in the evening, coinciding with *ELF3* expression [72,91]. However, *BBX18* expression peaks in the morning [91]. *BBX18* is a target of PRR5 [72,91] and interacts with PRR 5, 7, and 9 [73,91,145]. *XBAT31* is down-regulated in response to heat stress in the morning, when *BBX18* accumulates in response to heat stress [5]. *XBAT31* is unaffected by heat stress at night while *BBX18* remains at low levels and is not induced in the evening [5]. Identifying when the proposed interactions between XBAT31/35 and BBX18 occur under heat stress will require finer temporal resolution, examining the protein levels of XBAT31/35 and BBX18, and the activity of XBAT31/35. The lack of overlap in the transcriptional responses combined with changes in protein stability could provide a gating mechanism for thermosensing.

These studies demonstrate a role for ELF3 in sensing ambient changes in temperature (27-35°C) in Arabidopsis. It will be interesting to observe if the ELF3 PrD structure and subcellular localization influence the canonical heat-responsive gene expression network or if ELF3 plays a role in heat-stress response pathways at higher temperatures. PolyQ domains are not unique to plant circadian components; the animal *Clock* gene contains a PolyQ region [146], suggesting a fundamental relationship between circadian regulation and temperature sensing.

## Conclusions

- The combination of extrinsic and intrinsic factors complicates the study of elevated temperature responses in plants.
- Plants show time of day variation in their susceptibility to heat stress. The connections between heat stress response and circadian clock networks extend throughout the regulatory cascade. Therefore, considering the effects of the time of day when studying elevated temperature and heat stress responses is critical.
- ELF3 functions as a thermosensor regulating thermoresponsive growth. Increasing ambient temperatures affect ELF3 distribution in the cell. Two mechanisms have been proposed: (1) Increased temperatures alter ELF3 conformation leading to protein aggregation, or (2) warmer temperatures destabilize the EC routing ELF3 to proteasome degradation.

## Perspectives

- New research demonstrates complex connections between increased temperatures, heat stress responses, and plant circadian systems that can enlighten our understanding of how changing climates will impact plants and improve the selection of genotypes with enhanced yields in future climates.
- From the function of ELF3 as a thermosensor to the overlapping regulation of heat stress and the circadian systems on every step of gene expression, the connections between circadian regulation and heat stress responses are pervasive in plants.
- Examining the interactions between elevated temperature response, heat stress, and circadian clock pathways may identify the mechanisms for emergent properties that coordinate the timing of temperature responses. Initial studies were in Arabidopsis under controlled environments. Therefore, research in field conditions is needed to ascertain how these factors interact to improve plant yields even in a changing climate.

## Abbreviations

CTD: carboxy-terminal domain
DREB2A: DEHYDRATION-RESPONSIVE ELEMENT BINDING PROTEIN 2A
EC: evening complex
HSFs: heat shock factors
HSPs: heat shock proteins
PIF7: PHYTOCHROME INTERACTING FACTOR7
